# Supplementary material for: The prevalence and associated factors of sleep deprivation among healthy college students in China: a cross-sectional survey
Source: PeerJ. 2023 Sep 18;11:e16009. doi: 10.7717/peerj.16009 (PMC10512935; doi:10.7717/peerj.16009)
Supplement: Supplemental Information 2 [file peerj-11-16009-s002.docx]

**Consent Details**

Instructions were set at the top of the questionnaire, including the main content of the questionnaire and voluntariness of participation. The participants continued to complete the questionnaire if they were interested and volunteered to participate in the study. And if not, the participants could quit the survey. The questionnaire was filled out anonymously. The participants did not sign written informed consent,

The instruction is below.

The questionnaire contains 3 parts: basic information, sleep and fatigue related questions. The survey is anonymous and does not involve personal information. If you are not interested in or do not want to participate in the survey, for whatever reason, you can turn off the web page and quit the survey. Thank you for your participation.
