# Supplementary figures and images for: The prevalence and associated factors of sleep deprivation among healthy college students in China: a cross-sectional survey
Source: PeerJ. 2023 Sep 18;11:e16009. doi: 10.7717/peerj.16009 (PMC10512935; doi:10.7717/peerj.16009)

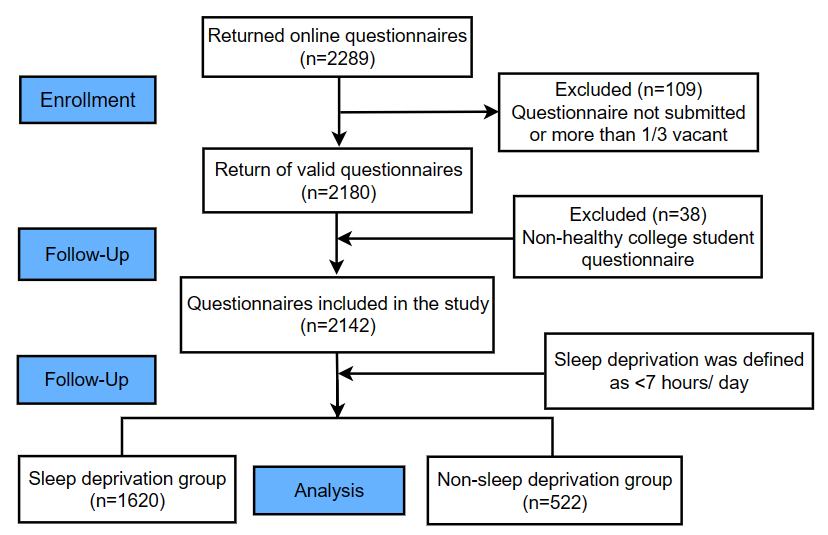

Supplement: Supplemental Information 3 [file peerj-11-16009-s003.png]
