# Supplementary material for: The prevalence and associated factors of sleep deprivation among healthy college students in China: a cross-sectional survey
Source: PeerJ. 2023 Sep 18;11:e16009. doi: 10.7717/peerj.16009 (PMC10512935; doi:10.7717/peerj.16009)
Supplement: Supplemental Information 4 [file peerj-11-16009-s004.docx]

**Supplement Table1.** Sleep characteristics of health and non-health students

|  | **Health（N=2142）** | **Non-Health（N=38）** | **t or χ^2^** | **P** |
| --- | --- | --- | --- | --- |
| PSQI total score (Median±SD) | 5.90±3.21 | 3.39±3.89 | -5.509 | 0.000*** |
| Subjective sleep quality (Median±SD) | 1.02±0.75 | 1.63±0.71 | -4.987 | 0.000*** |
| Sleep latency (Median±SD) | 0.91±0.93 | 1.47±1.11 | -3.095 | 0.004** |
| Sleep duration (Median±SD) | 1.07±0.82 | 1.42±1.00 | -2.118 | 0.041* |
| Habitual sleep efficiency (Median±SD) | 0.60±0.85 | 0.58±0.83 | 0.141 | 0.888 |
| Sleep disturbances (Median±SD) | 0.80±0.55 | 1.39±0.76 | -4.795 | 0.000*** |
| Use of sleeping medication (Median±SD) | 0.03±0.23 | 0.47±1.03 | -2.667 | 0.011* |
| Daytime dysfunction (Median±SD) | 1.46±0.96 | 2.42±0.76 | -7.696 | 0.000*** |
| Sleep duration (Median±SD) | 7.56±0.84 | 7.03±1.19 | 3.774 | 0.000*** |
| ＜5h | 116 (5.4%) | 2 (5.3%) | 57.024 | 0.000*** |
| 5~6 h (contains 5 hours) | 449 (21.0%) | 2 (5.3%) |  |  |
| 6~7 h (contains 6 hours) | 1055 (49.3%) | 9 (23.7%) |  |  |
| 7~8 h (contains 7 hours) | 460 (21.5%) | 17 (44.7%) |  |  |
| ≥8 h | 62 (2.9%) | 8 (21.1%) |  |  |

*P＜0.05, ** P<0.01, *** P<0.001.
