# Supplementary material for: The prevalence and associated factors of sleep deprivation among healthy college students in China: a cross-sectional survey
Source: PeerJ. 2023 Sep 18;11:e16009. doi: 10.7717/peerj.16009 (PMC10512935; doi:10.7717/peerj.16009)
Supplement: Supplemental Information 5 [file peerj-11-16009-s005.docx]

**Statistical Reporting**

The statistical methods involved in this study include data normality test, descriptive statistics, chi-square test, analysis of covariance, and binary logistic regression.

1. The Kolmogorov-Smirnov one-sample test was performed to examine the normality of the data set. If the data conformed to normality then the t-test or ANOVA was used to compare the data between groups and were expressed as mean ± standard deviation, if the data did not conform to normality then the Mann-Whitney U test was used to compare the data between groups and were expressed as median and interquartile range.

**Test statistic：**The data on age, body mass index, and number of cigarettes smoked per day of the participants were not tested for normality.

|  | **Kolmogorov-Smirnov** | | |
| --- | --- | --- | --- |
|  | **The test statistic Z** | **df** | **p values** |
| Age | 0.197 | 2142 | 0.000 |
| Body mass index | 0.103 | 2142 | 0.000 |
| Number of cigarettes smoked per day by participants | 0.248 | 42 | 0.000 |

2. Descriptive statistics were used to describe the characteristics of the sample, such as the province of the participants, the average age, the proportion of males and females, education level, grade, nationality, and marital status. Furthermore, the participant's body mass index, smoking and drinking conditions were also described.

**Test statistic：** A total of 2,142 participants were obtained for analysis. A large proportion (55.4%, 1187) of participants was from Shandong, but the survey was completed by respondents in 27 out of the 34 China provinces. Of the 2,142 individuals, 1,549 (72.3%) were females, and 593 (27.7%) were males. The mean (SD) age was 18.9±2.3years (range=16-35 years). Junior college students made up the majority of the sample (59.4%), followed by undergraduates (34.9%). A considerable percentage of college students were in their freshman year (42.7%) or sophomore years (32.8%). Most of these students are ethnic Han (95.6%), and a few are of other ethnicities (4.4%). Almost all students were single (99.0%) and only a very few students were married (1.0%). Furthermore, few proportions of students reported smoking (2.0%) and drinking behavior (11.4%).

3. The chi-square test and the Mann-Whitney U test were used to compare the socio-demographics, sleeping characteristics, smoking and drinking conditions between groups of the two groups. In this study, participants were divided into two groups based on the National Sleep Foundation's sleep guidelines for adults: the participants who sleep <7 hours/day were assigned to the sleep deprivation group and the participants who sleep ≥7 hours/day were assigned to the non-sleep deprivation group.

**Test statistic：**According to whether they slept ＜7 hours, the subjects were divided into two groups: sleep deprivation and non-sleep deprivation. There were 1620 people in the sleep deprivation group and 522 people in the non-sleep deprivation group. Two groups compared socio-demographics and sleeping characteristics, found in the two groups（sleep-deprived students and non-sleep-deprived students）had significant differences in age (p＜0.001), nap duration (p＜0.001), and sleeping duration on workdays and rest days (p＜0.001).


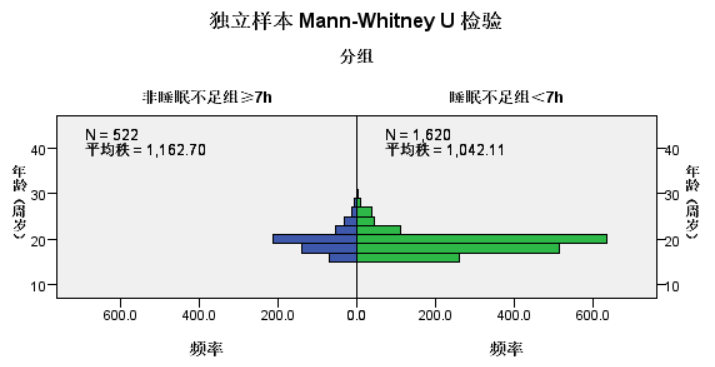


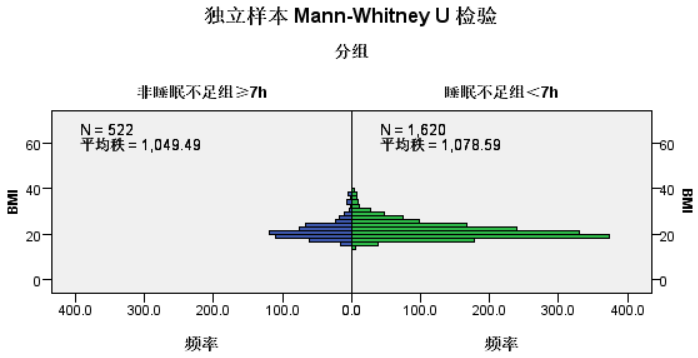


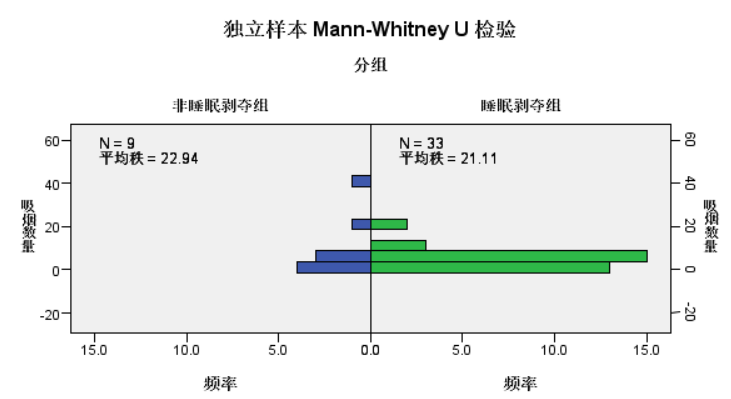


|  | **Pearson's chi-squared value** | **df** | **p values** |
| --- | --- | --- | --- |
| Education level | 4.998 | 2 | 0.082 |
| Grade | 9.442 | 5 | 0.092 |
| Gender | 0.709 | 1 | 0.400 |
| Marital status | 0.004 | 1 | 0.952 |
| Nationality | 3.274 | 6 | 0.783 |
| Smoking | 0.201 | 1 | 0.654 |
| Drinking | 0.749 | 1 | 0.387 |
| If drinking, frequency of drinking | 4.037 | 2 | 0.123 |
| If drinking, the amount of drunk in a day | 6.323 | 4 | 0.157 |
| Nap duration | 21.601 | 3 | 0.000 |
| Sleep duration on workdays and rest days | 6.398 | 2 | 0.040 |

4. An analysis of covariance was performed to observe socio-demographic, sleep, smoking and alcohol consumption characteristics of the sleep deprived and non-sleep deprived groups after controlling for age and gender as confounders.

**Test statistic：**Since age and gender were significantly associated with sleep duration, they were controlled as covariates and analyzed. The result showed that nap duration was also different between the sleep deprivation group and non-sleep deprivation group (F=9.007, p<0.01). However, there was no significant difference in BMI, education level, grade, marital status, nationality, smoking and drinking between sleep deprivation and non-sleep deprivation groups (all p>0.05) with/without adjusting for the effects of the age factor.

|  | **Adjusted R^2^** | **df** | **Mean Square** | **F** | **p values** |
| --- | --- | --- | --- | --- | --- |
| Education level | 0.525 | 2 | 0.632 | 1.465 | 0.321 |
| Grade | 0.525 | 4 | 0.526 | 1.218 | 0.301 |
| Marital status | 0.523 | 1 | 0.723 | 1.671 | 0.196 |
| Nationality | 0.523 | 6 | 0.288 | 0.665 | 0.678 |
| Smoking | 0.523 | 1 | 0.120 | 0.277 | 0.599 |
| Drinking | 0.523 | 1 | 0.256 | 0.591 | 0.442 |
| Nap duration | 0.529 | 3 | 2.047 | 4.791 | 0.002 |
| Sleep duration on workdays and rest days | 0.524 | 2 | 0.553 | 1.280 | 0.278 |

5. Binary logistic regression was used to predict risk factors and protective factors for sleep deprivation. Binary logistic regression was used to predict risk factors and protective factors for sleep deprivation. The accuracy of the results after adjusting for the independent variables was higher than that of the unadjusted results.

**Test statistic:** In adjusted models, sleep deprivation was associated with age, nap duration, sleeping duration on work and rest days, accustomed to staying up late, staying up late to work or study, stressed out state, repeating thoughts in bed.

| **Unadjusted models** | | |
| --- | --- | --- |
|  | df | **p values** |
| Age | 1 | 0.001 |
| Education level | 2 | 0.085 |
| Grade | 5 | 0.097 |
| Gender | 1 | 0.400 |
| Marital status | 1 | 0.952 |
| Nationality | 6 | 0.760 |
| Smoking | 1 | 0.654 |
| Drinking | 1 | 0.387 |
| Nap duration | 3 | 0.000 |
| Sleep duration on workdays and rest days | 2 | 0.042 |
| BMI | 1 | 0.324 |
| Taking time away from sleep for recreation | 1 | 0.515 |
| Drinking refreshing drinks (such as coffee, tea and others) | 1 | 0.820 |
| Accustomed to staying up late | 1 | 0.000 |
| Staying up late to work or study | 1 | 0.000 |
| The dormitory environment affects sleep | 1 | 0.006 |
| Relationship issues | 1 | 0.012 |
| Stressed out state | 1 | 0.000 |
| Repeated thoughts in bed | 1 | 0.002 |
| Mood problems | 1 | 0.006 |
| Desire to coordinate multiple life roles | 1 | 0.044 |

| **Adjusted** **models** | | |
| --- | --- | --- |
|  | df | **p values** |
| Age | 1 | 0.025 |
| Education level | 2 | 0.082 |
| Grade | 5 | 0.093 |
| Gender | 1 | 0.400 |
| Marital status | 1 | 0.952 |
| Nationality | 6 | 0.745 |
| Smoking | 1 | 0.654 |
| Drinking | 1 | 0.387 |
| Nap duration | 3 | 0.000 |
| Sleep duration on workdays and rest days | 2 | 0.041 |
| BMI | 1 | 0.324 |
| Taking time away from sleep for recreation | 1 | 0.515 |
| Drinking refreshing drinks (such as coffee, tea and others) | 1 | 0.820 |
| Accustomed to staying up late | 1 | 0.000 |
| Staying up late to work or study | 1 | 0.000 |
| The dormitory environment affects sleep | 1 | 0.140 |
| Relationship issues | 1 | 0.136 |
| Stressed out state | 1 | 0.000 |
| Repeated thoughts in bed | 1 | 0.002 |
| Mood problems | 1 | 0.348 |
| Desire to coordinate multiple life roles | 1 | 0.310 |
