# Supplementary material for: The prevalence and associated factors of sleep deprivation among healthy college students in China: a cross-sectional survey
Source: PeerJ. 2023 Sep 18;11:e16009. doi: 10.7717/peerj.16009 (PMC10512935; doi:10.7717/peerj.16009)
Supplement: Supplemental Information 6 [file peerj-11-16009-s006.docx]

**Supplemental Statistical Reporting**

The statistical methods involved in the supplementary tables of this study include the chi-square test and t-test.

1. The chi-square test and t-test were used to compare the sleeping characteristics between groups of health and non-health.

Test statistic: According to the questions for health status determination answered by the participants in the questionnaire, the participants were divided into health and non-health groups. There were 2,142 people in the health group and 38 people in the non-health group. Two groups compared sleeping characteristics found the two groups (the health group and the non-health group）had significant differences in PSQI total score (p＜0.001), subjective sleep quality (p＜0.001), sleep latency (p＜0.01), sleep duration (p＜0.05), sleep disturbances (p＜0.001), use of sleeping medication (p＜0.05), daytime dysfunction (p＜0.001), sleep duration (p＜0.001).

|  | **Pearson's chi-squared value** | **df** | **p values** |
| --- | --- | --- | --- |
| Sleep duration | 57.024 | 4 | 0.000 |
| <5 h |  |  |  |
| 5~6 h (contains 5 hours) |  |  |  |
| 6~7 h (contains 6 hours) |  |  |  |
| 7~8 h (contains 7 hours) |  |  |  |
| ≥8 h |  |  |  |

|  | **t-test** | **df** | **p values** |
| --- | --- | --- | --- |
| PSQI total score | -5.509 | 2178 | 0.000 |
| Subjective sleep quality | -4.987 | 2178 | 0.000 |
| Sleep latency | -3.095 | 37.923 | 0.004 |
| Sleep duration | -2.118 | 37.871 | 0.041 |
| Habitual sleep efficiency | 0.141 | 2178 | 0.888 |
| Sleep disturbances | -4.795 | 37.708 | 0.000 |
| Use of sleeping medication | -2.667 | 37.063 | 0.011 |
| Daytime dysfunction | -7.696 | 39.129 | 0.000 |
| Sleep duration | 3.774 | 2178 | 0.000 |

2. The supplementary figure shows the results of prevalence and the factors of sleep deprivation among Chinese college students of the present study. The data are based on and summarize the results of the full study.
